# QuickStats

**Published:** 2014-03-28

**Authors:** FROM THE NATIONAL CENTER FOR HEALTH STATISTICS

**Figure:**
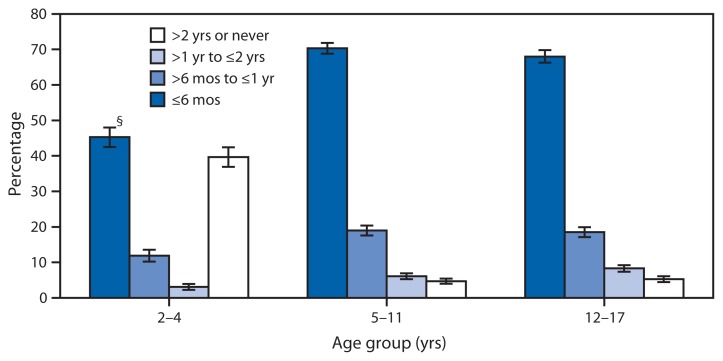
Time Since Last Dental Visit^*^ by Children Aged 2–17 Years, by Age Group — National Health Interview Survey,^†^ United States, 2012 ^*^ Based on response to the question, “About how long has it been since [child’s name] last saw a dentist? Include all types of dentists, such as orthodontists, oral surgeons, and all other dental specialists, as well as dental hygienists.” ^†^ Estimates were based on household interviews of a sample of the noninstitutionalized U.S. civilian population and are derived from the National Health Interview Survey sample child component. ^§^ 95% confidence interval.

During 2012, approximately 69% of children aged 5–17 years had a dental visit in the past 6 months; among children aged 2–4 years, the percentage with a dental visit was 45%. Approximately 19% of those aged 5–17 years and 12% of those aged 2–4 years had a visit >6 months to ≤1 year before. Approximately 40% of those aged 2–4 years and 5% of those aged 5–17 years had not had a dental visit in >2 years or had never seen a dentist.

**Source:** Bloom B, Jones LI, Freeman G. Summary health statistics for U.S. children: National Health Interview Survey, 2012. Vital Health Stat 2013;10(258).

**Reported by:** Lindsey Jones, MPH, izf4@cdc.gov, 301-458-4548; Barbara Bloom, MPA; Gulnur Freeman, MPA.

